# Lipidomics by Nuclear Magnetic Resonance Spectroscopy and Liquid Chromatography–High-Resolution Mass Spectrometry in Osteosarcoma: A Pilot Study

**DOI:** 10.3390/metabo14080416

**Published:** 2024-07-28

**Authors:** João Guilherme de Moraes Pontes, Milka Jadranin, Márcia Regina Assalin, Melissa Quintero Escobar, Danijela Stanisic, Tássia Brena Barroso Carneiro Costa, André van Helvoort Lengert, Érica Boldrini, Sandra Regina Morini da Silva, Daniel Onofre Vidal, Leticia Huan Bacellar Liu, Mariana Maschietto, Ljubica Tasic

**Affiliations:** 1 Laboratory of Biological Chemistry, Institute of Chemistry, Universidade Estadual de Campinas, Campinas 13083-970, Brazil; jgpontes@unicamp.br (J.G.d.M.P.); milka.jadranin@ihtm.bg.ac.rs (M.J.); marcia.assalin@embrapa.br (M.R.A.); meliquies@gmail.com (M.Q.E.); danijela@unicamp.br (D.S.); tassia.costa@iqb.ufal.br (T.B.B.C.C.); l172226@dac.unicamp.br (L.H.B.L.); 2 Department of Chemistry, Institute of Chemistry, Technology and Metallurgy, University of Belgrade, Njegoševa 12, 11000 Belgrade, Serbia; 3 Embrapa Environment, Jaguariúna 13820-000, Brazil; 4 Molecular Oncology Research Center (CPOM), Barretos Cancer Hospital, Barretos 14784-400, Brazil; ahlengert@gmail.com (A.v.H.L.); danielovidal@gmail.com (D.O.V.); 5 Barretos Children’s Cancer Hospital, Barretos 14784-400, Brazil; boldrinierica@gmail.com; 6 Department of Pathology, Barretos Cancer Hospital, Barretos 14784-400, Brazil; smmorini61@gmail.com; 7 Brazilian Biosciences National Laboratory (LNBio), Brazilian Center for Research in Energy and Materials (CNPEM), Campinas 13083-100, Brazil; marianamasc@gmail.com

**Keywords:** bone tumor, osteosarcoma, metastasis, nuclear magnetic resonance spectroscopy, mass spectrometry

## Abstract

Cancer is a complex disease that can also affect the younger population; however, it is responsible for a relatively high mortality rate of children and youth, especially in low- and middle-income countries (LMICs). Besides that, lipidomic studies in this age range are scarce. Therefore, we analyzed blood serum samples from young patients (12 to 35 years) with bone sarcoma (osteosarcoma) and compared their lipidomics to the ones from the control group of samples, named healthy control (HC group), using NMR and LC-MS techniques. Furthermore, differences in the lipidomic profiles between OS patients with and without metastasis indicate higher glycerophosphocholine (GPC) and glycerophospholipid (GPL) levels in osteosarcoma and increased cholesterol, choline, polyunsaturated fatty acids (PUFAs), and glycerols during the metastasis. These differences, detected in the peripheral blood, could be used as biomarkers for liquid biopsy.

## 1. Introduction

Cancer is a complex disease that alters a cell’s metabolism during its initiation and progression [1]. There are an estimated 400,000 new cases of cancer globally each year in children and adolescents aged 1 to 19 years [2]. As many as two-thirds of childhood cancer survivors will experience complications related to cancer and its treatment received via chemotherapy and/or radiotherapy that may adversely affect quality of life and increase the risk of premature death [3,4]. Thus, research on pediatric tumors is essential both for a better understanding of their biology, development, and progression and to facilitate the discovery and development of treatment with fewer secondary effects [5]. Some studies have revealed the potential role of metabolomics/lipidomics in gaining an understanding of pathophysiological processes in cancer, improving tumor staging, characterizing tumors, and searching for biomarkers predictive of therapeutic responses [6,7,8]. However, it is necessary to have a better understanding of how these measurements are associated with human physiology and cancer disease [8]. In this sense, lipids have been highlighted in cancer research since this compound class plays an important role in membrane structure, energy storage, and signal transduction [7]. The investigation of lipid biochemistry using a lipidomic approach can provide insights into the specific roles of lipid molecular species in health and disease, allowing for the identification of lipid-related pathways that are altered in various physiological conditions. Further, lipid biochemistry can assist in the identification of potential biomarkers for establishing preventive or therapeutic approaches for human health [9,10].

Osteosarcoma (OS) is the most common primary malignant bone tumor, which can affect children, adolescents, and young adults and causes pain and swelling besides other symptoms such as mobility and weight loss, restricting the life of the patient [11]. In current research related to this issue, different biochemical alterations have been reported such as changes in gene expression [12,13,14]; proteins [15,16]; and metabolites related to arginine, glutathione, inositol, and fatty acid metabolic pathways [6]. 

Previously, in a reported metabolomics study, alterations in lipids, aromatic amino acids, and histidine levels were observed [6]. Therefore, we aimed to gain a better understanding of the role of lipids in OS. Herein, we performed lipidomic studies of blood from youth patients diagnosed with osteosarcoma using nuclear magnetic resonance (NMR) spectroscopy and mass spectrometry techniques. These results provide a better understanding of altered mechanisms related to osteosarcoma and disclose potential biomarkers for diagnosis and disease monitoring.

## 2. Materials and Methods

### 2.1. Blood Serum Samples and Lipid Extracts

Twenty-one blood serum samples of patients diagnosed with osteosarcoma stored at the biobank from Hospital do Amor (Barretos, Sao Paulo, Brazil) were selected. Seventeen patients presented complete clinical and pathological information, as depicted in Table S1 (Supplementary Materials), while four patients’ data were not available. Blood samples collected from healthy individuals were used as a control group (8 samples, Table S2, Supplementary Materials). These samples have been used in previous studies [6]. The serum samples were thawed at room temperature and centrifuged at 3939× *g* for 15 min at 4 °C. Each serum sample was stored at −80 °C until lipid extraction before NMR and LC-MS analyses.

Lipids were obtained as dissolved in a chloroform phase after extraction by applying a chloroform-methanol mixture (2:1, *v*/*v*) on serum samples. The extraction process was performed with ice-cold solvents and lasted around 40 min. The solvent was removed by rota-evaporation, and lipids were dried in the mild nitrogen stream. Lipids were used as fresh samples following sample preparation for NMR or MS analyses or dissolved in the appropriate solvent and kept at 4 °C until analyses.

### 2.2. NMR Spectra Acquisition

The procedure for sample preparation and NMR spectra acquisition is based on previous reports [17,18]. Lipids were dissolved in 600 μL of chloroform-*d* (CDCl_3_ with 0.03% (*v*/*v*) of tetramethylsilane (TMS), Sigma-Aldrich, Burlington, MA, USA) at room temperature, and transferred into 5 mm NMR tubes. High-resolution ^1^H-NMR (*zg30*) spectra were acquired on the Bruker AVANCE III 600 MHz spectrometer using the inverse triple-core probe (TBI) at 25 °C. The acquisitions were performed with 128 scans, relaxation delay of 1 s, acquisition time of 2097 s, receptor gain of 181, free-induction decay size of 65,536, and 13.02 ppm spectral width for 1D spectra. The two-dimensional experiments (Heteronuclear Single-Quantum Coherence-^1^H,^13^C-HSQC, and Heteronuclear Multiple-Bond Correlation-^1^H,^13^C-HMBC) were performed on randomly selected samples.

All 1D NMR spectra were phased and baseline-corrected, and the chemical shifts were referenced to TMS (δ 0.00). The NMR spectra of lipid extract samples were processed using MestReNova software (14.0.1-23559). Samples were normalized to a constant sum (100) of the entire spectra intensity to reduce the differences in concentration. After that, the spectra were divided into regions with equal widths of 0.001 ppm (bins) and used to construct the matrix for multivariate analysis.

### 2.3. Statistical Analysis of NMR Data

Spectral data obtained by processing and normalization of NMR spectra were transported into the matrix lines and organized into a single matrix containing the samples (cases) in the columns and the bins in the lines (variables). The matrices were submitted to the chemometric analyses and analyzed using the MetaboAnalyst 5.0 software platform [19].

The chemometrics matrix was constructed using δ 0.20–6.80 of the ^1^H-NMR spectra for osteosarcoma patients, including data from patients with metastasis at diagnosis, and for healthy control (HC) patients, amounting to a total of 2951 variables. These data were modeled with the supervised method of partial least-square–discriminant analysis (PLS-DA) to discover the metabolite differences between the groups. According to the PLS-DA models, the highest values from the variable importance in projection (VIP) scores were used to depict the most significant chemical shifts for each class. Leave-one-out cross-validation (LOOCV) was performed, and confusion matrices were constructed to evaluate the classification models. Accuracy, specificity, and sensitivity values were commuted and analyzed.

The metabolites were assigned based on chemical shifts, coupling constants, and databases, namely The Human Metabolome Database (HMDB, ref. [20] and the Biological Magnetic Resonance Data Bank (BMRB, ref. [21]).

### 2.4. LC-MS Analysis of Lipid Extracts

For untargeted lipidomic analysis, 25 μL volumes of chloroform solutions of lipids separated for LC-MS analysis were dissolved in 975 μL of water–isopropanol mixture (prepared from 1 mL of water and 2-propanol (LC-MS grade) until the volume reached 25 mL in a volumetric flask) and injected into an ultra-high performance liquid chromatography instrument (Waters Acquity Ultra Performance LC; Milford, MA, USA) connected to a hybrid quadrupole orthogonal time-of-flight (Q-ToF) mass spectrometer (Waters Synapt HDMS; Milford, MA, USA) equipped with an electrospray ion source, and MassLynx software version 4.1 (Waters Corp., Milford, MA, USA). The separation of lipid compounds was performed using an Acquity UPLC BEH C18 column (100 mm × 2.1 mm; 1.7 μm, Waters). The mobile phase was composed of solvent A (water–acetonitrile (60:40, *v*/*v*)) and solvent B (isopropanol–acetonitrile (90:10, *v*/*v*)); both solvents contained 10 mmol L^−1^ ammonium formate and 0.1% formic acid. The following gradient program was used: 0–2 min 40–43% B, 2–2.1 min 43–50% B, 2.1–12 min 50–54% B, 12–12.1 min 54–70% B, 12.1–18 min 70–99% B, 18.0–18.1 min 99–40% B, and 18.1–20 min 40% B. The mobile-phase flow rate was 0.40 mL min^−1^, the column temperature was 55 °C, and the injection volume of samples and blanks was 5 μL. Positive ion mode was recorded in the *m*/*z* range of 50–1000, under the following conditions: capillary voltage, 2.60 kV; cone voltage, 40 V; source temperature, 120 °C; desolvation gas temperature (nitrogen), 450 °C; and desolvation gas flow (nitrogen), 500 L/h. Sodium formate solution (10% formic acid solution/0.1 mmol L^−1^ sodium hydroxide solution/acetonitrile, 1:1:8, *v*/*v*/*v*) was used to calibrate the mass spectrometer (within the scope of 50 to 1000 Da) and as the external reference of Lock Spray TM *m*/*z* 566.8891 in positive ion mode, which was injected at a continuous flow of 10 μL min^−1^. Samples were randomly analyzed and local quality-control samples, prepared by pooling aliquots from all serum specimens of each group, were injected after every ten injections to monitor system stability. A blank sample, prepared by dissolving 25 μL of chloroform in 975 μL of the water–isopropanol mixture, was injected between every two samples of lipid extracts.

### 2.5. LC-MS Data Processing and Statistical Analysis

The raw data from Waters (RAW) were converted to mzXML data format for peak picking using MSConvert software version 3.0 (https://proteowizard.sourceforge.io/ accessed on 20 July 2024) [22]. Peak detection and retention-time alignment were performed using the XCMS online platform within the R statistical programming environment [23,24,25]. For the collected data, XCMS parameters optimized for Waters QToF instruments include centWave feature detection, orbitrap retention-time correction, the minimum fraction of samples in one group to be a valid group = 0.50, *p*-value thresholds for patients versus control samples = 0.05, isotopic ppm error = 15, width of overlapping *m*/*z* slices (mzwid) = 0.010, bandwidth grouping (bw) = 2, minimum peak width = 2 s, and maximum peak width = 25 s. The resulting peak table comprising retention times, *m*/*z* values, and peak intensities was exported for further processing and organized into a single matrix containing the samples (cases) in the columns and the *m*/*z* values in the lines (variables), with the division of a column referring to the classification of the samples (class variable: OS—osteosarcoma patients and HC—healthy control). The matrix was constructed with 44 chromatograms (32 for the osteosarcoma group and 12 for the HC group) and 140 variables and submitted to the subsequent chemometric analyses performed using the MetaboAnalyst platform version 6.0 (www.metaboanalyst.ca accessed on 20 July 2024) [19]. The metabolites were assigned based on accurate mass measurements reported in the literature and comparison with databases HMDB [20] and LipidMaps (LMSD) [26].

## 3. Results and Discussion

### 3.1. NMR-Based Lipidomics of Osteosarcoma

**Figure 1:**
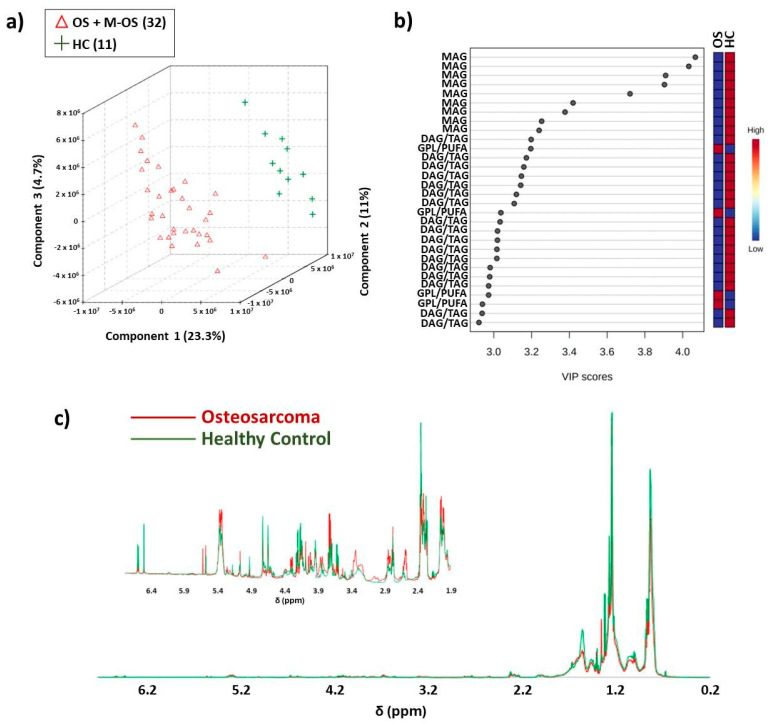
Illustration of the most significant NMR lipidomic results. The PLS-DA obtained for osteosarcoma with and without metastasis (OS + M-OS) patients (red color) and healthy control (HC, green color): (**a**) score plot using spectral region between δ 0.20 and 6.80, with 23.3% variance in PC 1, 11.0% in PC 2 and 4.7% in PC 3; (**b**) VIP values generated by PLS-DA model; (**c**) overlap of ^1^H-NMR (δ 0.20–6.80) mean spectra of the lipids and above; the overlap of ^1^H-NMR (δ 1.90–6.50) mean spectra with increased intensity. Chemometrics results were obtained on the MetaboAnalyst platform. OBS: Samples were analyzed in duplicate; however, not all data were used for PLS-DA.

The results of the partial least-square–discriminant analysis (PLS-DA), which was applied to blood serum lipids found in the NMR range between δ 0.00 and 6.80 (Figure 1), indicated discrimination between osteosarcoma patients and the control group, with an accuracy of 0.953, *Q*^2^ of 0.658, and *R*^2^ of 0.927, using three components. Examples of ^1^H-NMR spectra acquired from HC and Osteosarcoma samples are available in Figure S1 in the Supplementary Materials.

**Figure 2:**
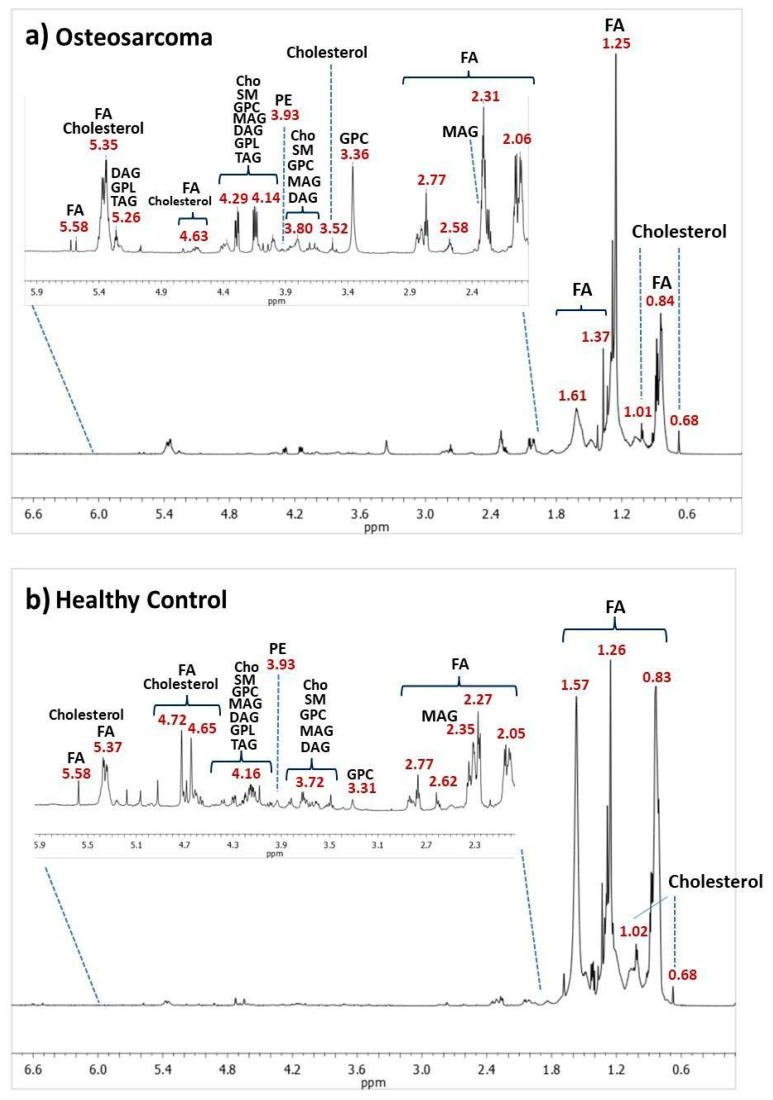
^1^H-NMR spectra (δ 0.00–6.80) acquired using *zg30* pulse sequence: (**a**) representative serum lipid extracts of OS patients and (**b**) healthy control. Abbreviations: Cho, choline; DAG, diacylglycerol; FA, fatty acyls; GPC, glycerophosphocholine; GPL, glycerophospholipid; MAG, monoacylglycerol; PE, phosphatidylethanolamine; SM, sphingomyelin; TAG, triacylglycerol. A more detailed assignment of the peaks of ^1^H-NMR spectra is shown in Table S3 in the Supplementary Materials.

Analyzing the VIP scores (Figure 1b), it was found that δ 2.55–2.58 and δ 2.79 were important variables with higher intensity in OS class, which distinguished the two groups, while variables at δ 2.35, 3.59, 3.62, 4.18, and 4.73 were important for the classification and showed higher intensities in the HC class. A visual inspection of the ^1^H-NMR mean spectra (Figure 1c) indicated more intense peaks in the OS spectral regions at δ 1.99–2.06, 2.55–2.62, 2.85–2.96, 3.22–3.37, 3.70–3.90, 4.28–4.40, 5.32-5.40, and 5.53–5.62 and lower intensity at δ 0.83–1.57, 2.35–2.39, 3.59–3.62, 4.15–4.23, 4.60–4.74, 4.93, 6.50, and 6.59. Peak assignments are shown in Figure 2.

A thorough analysis of the ^1^H-NMR spectral and VIP score data indicated a higher intensity of peak at δ 2.35 and 4.18 in HC samples, assigned to monoacylglycerols (MAG) [27]. Many studies have reported a monoacylglycerol lipase (MAGL) overexpression in cancer cells [28,29]. Furthermore, previous studies have indicated that MAGL may play a biological role in attenuating osteosarcoma growth and metastasis (28). Therefore, a decrease in MAG levels in OS patients could be due to the MAGL excess, since it is acting in defense processes and catabolizing all the MAG available in the biological system into glycerol and free fatty acids [30,31]. The same could be happening with triacylglycerols (TAGs) since MAGL also decomposes these compounds [28]. Our results indicate a higher intensity of peaks between δ 4.15 and 4.23 in the HC group, which corroborates with assignments for TAG reported in the literature [32].

Changes in the NMR peak intensities assigned to diacylglycerols (DAGs) were also observed (δ 3.72, 4.16, 5.07) in OS [33]. DAG levels regulate cell growth and differentiation, and DAGs can be converted to phosphatidic acids mediated by diacylglycerol kinases (DGKs) [34,35], which corroborates with higher NMR peak intensities at δ 4.16 and 5.07 in the HC group in our results. Currently, diacylglycerol kinase zeta (DGKZ) is reported as a potential gene associated with specific human carcinogenesis [34]. We also observed increases in the intensities of assigned peaks to glycerophosphocholine (GPC) and choline (Table S3, Figure 1c) in OS patients’ spectra, which corroborates with previous studies that reported using osteosarcoma cell samples and in other cancer types [36,37]. GPC is one important precursor for phosphatidylcholines (PCs), a membrane lipid. Perturbations in phospholipid metabolism have been reported in apoptosis processes [38]. The activation of the choline metabolism has been reported as a critical step in the progression of different cancer types, which leads to the increase in choline-containing compounds [39].

Other NMR-increased peak intensities observed in osteosarcoma samples and important for class discrimination (Figure 1b) were noted at δ 2.55 and 2.79 and assigned to polyunsaturated fatty acids (PUFAs) [40] and glycerophospholipids (GPLs) [41]. PUFA accumulation has been reported as a potential biomarker of cell ferroptosis in osteosarcoma since iron participates in the lipid hyperoxidation process [42]. Alterations in glycerophospholipid metabolic pathways have been previously reported in OS research, which indicated a higher differentiation in the lecithin–cholesterol acyltransferase (LCAT) gene and some GPLs, including phosphoethanolamine [43,44].

**Figure 3:**
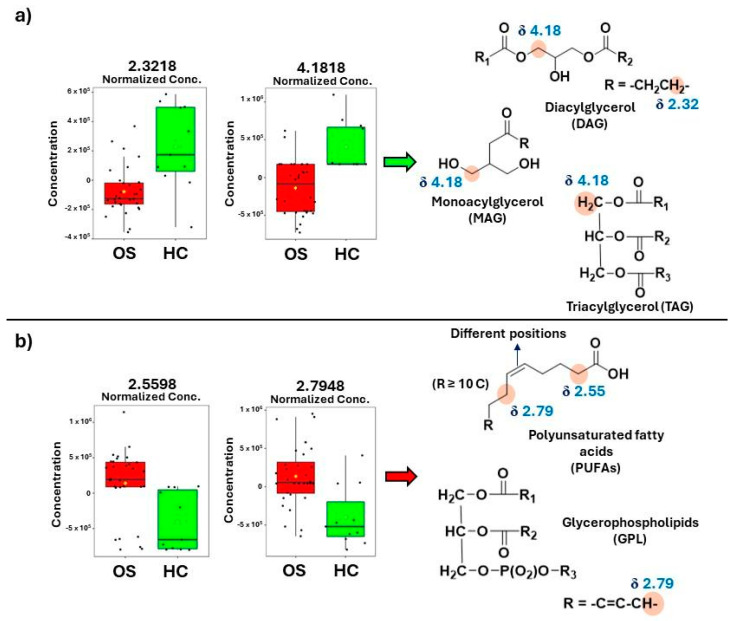
Box plots obtained in MetaboAnalyst showing the normalized concentration of some important variables for the two classes’ separation: (**a**) increased metabolite levels in healthy control (HC, green color); (**b**) increased metabolite levels in osteosarcoma patients (OS, red color).

An increase in NMR peak intensities between δ 3.77 and 3.83 was assigned to cholesterol. Cholesterol is a lipid component of cell membranes, together with GPLs and sphingolipids [45]. Cholesterol was previously reported as a biomarker in osteosarcoma prognosis since differentiation was observed in glycolysis and cholesterol synthesis-related genes (GCSRGs) per tumor subtype and its microenvironments [46]. Figure 3 illustrates box plots of important OS and HC classification variables.

### 3.2. Differentiation between Osteosarcoma Patients with and without Metastasis

Metastasis affects the biological system in different ways, leading to biochemical changes such as altered metabolic pathways, cellular behavior in the face of stimuli, and responses to medications such as chemotherapy and radiation therapy [47]. Approximately 20% of the patients present metastasis at initial diagnosis, and more than 80% of the cases occur with lung metastasis [48]. Currently, there is no specific laboratory test for osteosarcoma, so the search for biomarkers is one of the strategies that have been elaborated for this purpose [49].

**Figure 4:**
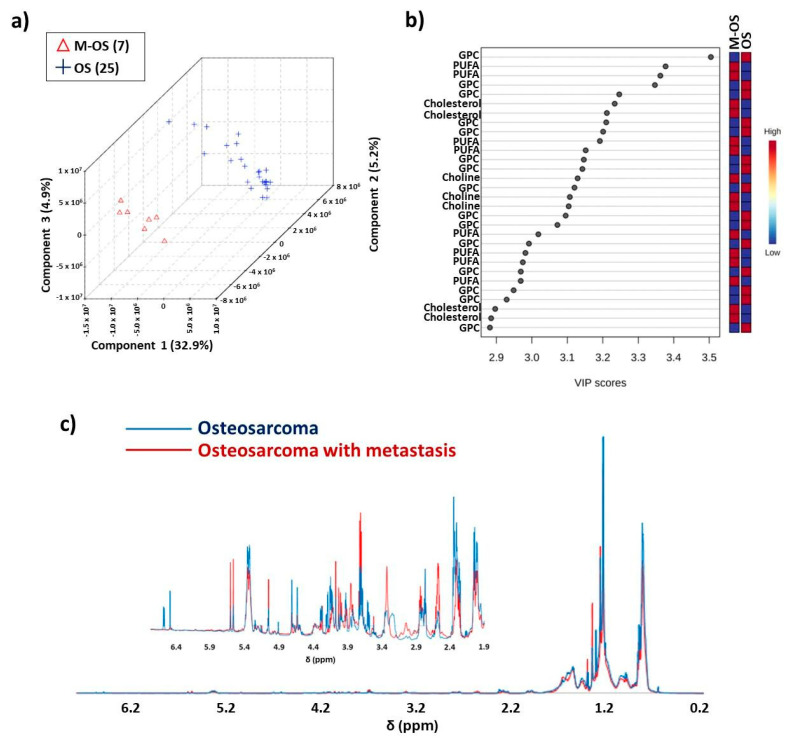
Illustration of classification results for the PLS-DA obtained for osteosarcoma patients with metastasis (M-OS, red color) and without metastasis (OS, blue color) at diagnosis: (**a**) score plot using spectral region between δ 0.20 and 6.80, with 32.9% variance in PC 1, 5.2% in PC 2 and 4.9% in PC 3; (**b**) VIP values generated by PLS-DA model; (**c**) overlap of the ^1^H-NMR (δ 0.20–6.80) mean spectra of the lipid extracts and above; the overlap of ^1^H-NMR (δ 1.90–6.50) mean spectra with increased intensity. Chemometrics results were obtained using the MetaboAnalyst platform. OBS: Samples were analyzed in duplicate; however, not all data were used in PLS-DA.

A thorough analysis of the PLS-DA (Figure 4) results showed the classification of the samples between osteosarcoma patients who suffered metastasis (M-OS) and with the absence of metastasis (OS), with discrimination accuracy of 0.844, *Q*^2^ of 0.107, and *R*^2^ of 0.970, using three components.

An analysis of the VIP scores (Figure 4b) was indicative of δ 2.60, 3.32, 3.52, 3.87, and 5.61 as variables with higher intensity in the M-OS class, which were important to distinguish the two groups, while variables at δ 3.21, 3.25, 3.59, and 3.60 were important and showed higher intensities in the OS class. The ^1^H-NMR mean spectra (Figure 4c) indicated more intense peaks in the M-OS spectral regions at δ 2.50–2.60, 2.80–2.89, 3.22–3.39, 3.70–3.90, 5.10, and 5.53–5.62, while higher intensity of peaks in OS spectral regions were at δ 0.83, 1.20–1.22, 1.90–2.40, 4.60–4.74, 5.38–5.40, 6.50, and 6.59.

Variables δ 2.60 and 5.61 in VIP scores (Figure 4b) were higher in the M-OS group, in which respective chemical shifts were assigned to PUFAs. These data corroborate previous research, which has reported that eicosanoids play an important role in cell growth and metastasis since they act as inflammatory mediators [50]. Another metabolite annotated with elevated concentrations in the M-OS group was choline (δ 3.52). This metabolite was previously reported as a potential biomarker for differentiation between benign and malignant bone tumors [51]. However, other studies indicated that the elevation in choline levels may vary amongst tumors [52]. The increase in choline levels could be due to the overexpression of phospholipase D commonly found in cancer development [51,53], which helps in the conversion of phosphatidylcholines to choline [54].

An increased NMR peak intensity between δ 3.77 and 3.83 was observed in osteosarcoma spectra, which was assigned to cholesterol (Figure 4c). Elevated cholesterol levels have been reported as a metastatic biomarker in different cancer types, including osteosarcoma [55,56]. A hypothesis for the cholesterol accumulation during the metastasis process could be caused by a decrease in carbohydrates and amino acid metabolism and the upregulation of lipid metabolism [57].

**Figure 5:**
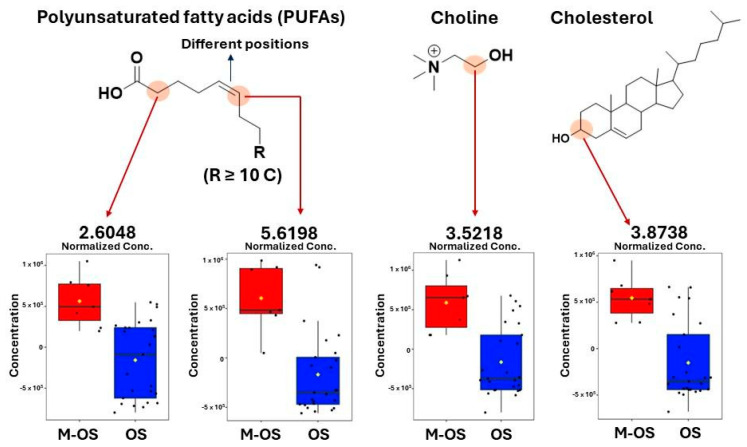
Box plots obtained in MetaboAnalyst showing the normalized concentration of some important variables for the two classes; separation, namely osteosarcoma patients with metastasis (M-OS, red color) and without metastasis (OS, blue color).

A higher concentration of glycerophospholipids (δ 3.21 and 3.60) in OS patients without metastasis was observed. The reduction in GPLs during the metastatic process is likely related to the increase in cytosolic phospholipase A2 (cPLA2) overexpression, which catalyzes the hydrolytic reaction of GPLs, producing lysophospholipids and fatty acids [58,59]. Figure 5 illustrates box plots of variables important for the two classes’ separation (with and without metastasis).

### 3.3. ESI (+) LC-MS-Based Lipidomics of Osteosarcoma

**Figure 6:**
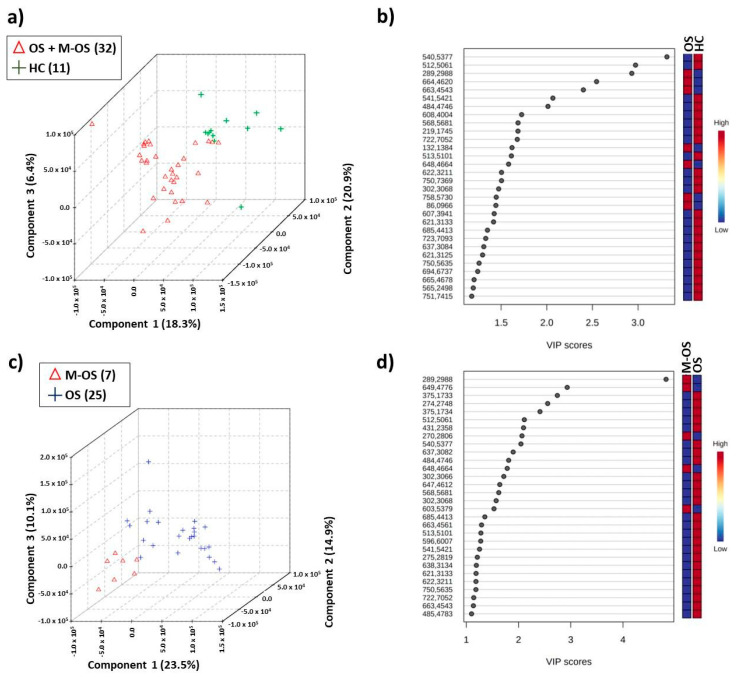
PLS-DA of the ESI(+) LC-MS chromatograms using osteosarcoma (OS) samples, osteosarcoma with metastasis (M-OS), and healthy control (HC): (**a**) score plot in OS + M-OS (red color) × HC (green color) analysis with 18.3% variance in PC 1, 20.9% in PC 2, and 6.4% in PC 3; (**b**) VIP scores generated in OS × HC analysis; (**c**) score plot in M-OS (red color) × OS (blue color) analysis with 23.5% variance in PC 1, 14.9% in PC 2, and 10.1% in PC 3; (**d**) VIP scores generated in M-OS × OS analysis.

Multivariate analysis (Figure 6) was performed with LC-MS data and pointed to the discriminant *m*/*z* values of lipidic compounds that contributed to the class separation. The examples of the total ion chromatograms (TICs) obtained by ESI (+) LC-MS for lipids of osteosarcoma patients and healthy controls are shown in Figure S2 in the Supplementary Materials.

Investigating the results obtained in the PLS-DA was indicative of the discrimination between osteosarcoma patients and the control group, with an accuracy of 0.750, *Q*^2^ of 0.12027, and *R*^2^ of 0.3908, using a one-component model, as well as discrimination between osteosarcoma with metastasis and without metastasis, with an accuracy of 0.71875, *Q*^2^ of 0.041696, and *R*^2^ of 0.80856 using four components. Table 1 shows assignments for the most important *m*/*z* features selected according to VIP scores (Figure 6b,d).

Ceramide (*m*/*z* 664.4620) was annotated as a possible biomarker of osteosarcoma. Previous studies reported the inhibition of ceramide glucosyltransferase (UGCG) in cancerous cells, which causes an increase in ceramide concentration, so it mediates the apoptosis process through mechanisms still not understood [63]. Another annotated potential biomarker for OS was the glycerophosphoethanolamine PE-NMe (18:1/18:1) (*m*/*z* 758.5730). This result corroborates with the NMR data, which indicated elevated levels of GPLs in the osteosarcoma group. Increased PE-NMe (18:1/18:1) levels have been reported in papillary thyroid cancer [64]. This lipid class plays important biological functions in cell membranes and is related to calcium (II) transport regulation in signaling [64,65].

In our results, *m*/*z* 648.4664 and 649.4776 were assigned to PA (18:1(9*Z*)/14:0) and PA (16:0/16:0), respectively. These lipids were important variables as they not only distinguished the OS group from the HC but were also altered during metastasis. PA (18:1(9*Z*)/14:0) and PA (16:0/16:0) are phosphatidic acids, and these metabolites have not been previously reported in osteosarcoma disease. However, increased phosphatidic acid levels have been associated with the autophagic process, which is necessary for tumor maintenance and the promotion of metastatic cascade [66,67]. Therefore, the increased phosphatidic acid levels during osteosarcoma could be due to the catabolism of DAGs by diacylglycerol kinases [34,35] and posteriorly in metastasis, due to the AMP-activated protein kinase (AMPK) suppression by phospholipase D1 (PLD1) in autophagy [66].

14-Methyleicos-en-1-yn-3-ol was annotated as a metabolite in higher concentration in osteosarcoma disease and metastasis. Fatty alcohols are commonly found in lysophosphatidic acids (LPAs) whose degradation may be a mechanism for LPA regulation [68,69].

**Figure 7:**
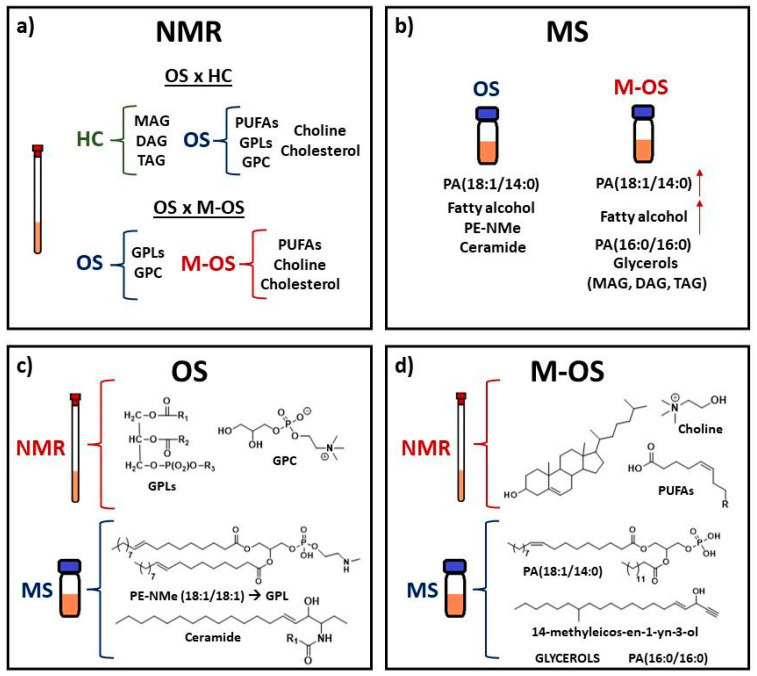
Increased concentration of metabolites for each group, namely healthy control (HC), osteosarcoma (OS), and osteosarcoma with metastasis (M-OS), following VIP scores and box plots: (**a**) potential biomarkers detected by NMR spectroscopy; (**b**) detected by LC-MS; (**c**) representative metabolites of the osteosarcoma group without metastasis and (**d**) with metastasis.

**Figure 8:**
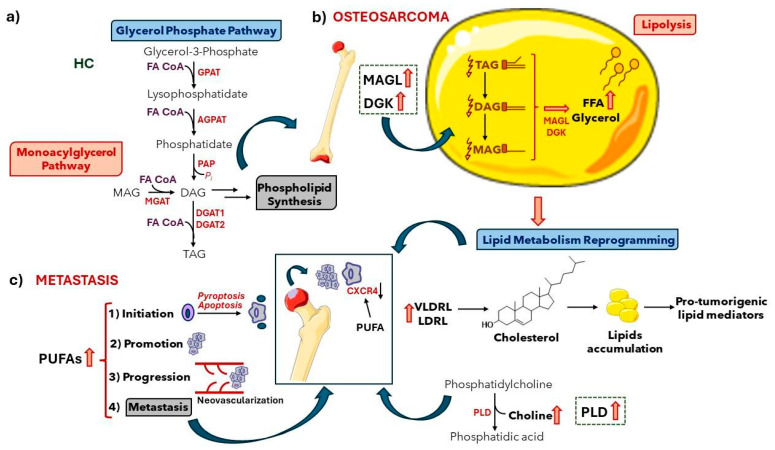
Biochemical pathways affected in osteosarcoma disease: (**a**) healthy control (HC); (**b**) osteosarcoma disease; (**c**) metastasis. Abbreviations: AGPAT, acyl-glycerol–phosphate acyltransferase; DGAT-acyl-CoA, diacylglycerol acyltransferase; GPAT, glycerol–phosphate acyltransferase; LDRL, low-density lipoprotein receptor; MGAT-acyl-CoA, monoacylglycerol acyltransferase; PAP, phosphatidic acid phosphohydrolase; VLDRL, very low-density lipoprotein receptor. OBS: Figures of bone and adipocyte were adapted from smart.servier.com (free medical images).

The variable *m*/*z* 603.5379 indicated an increase in intensity after the metastasis process (Figure 6d). The mass-to-charge ratio of 603.53 has been reported in the fragmentation pattern of DAGs and TAGs [60,61]. Although glycerols were found in higher intensity in HC samples (Figure 3) due to the increase in the expression of enzymes such as MAGL and DGK during cancer [28,29,34], the increase in DAG and TAG levels during metastasis could be a mechanism used to consume energy and release stored fatty acids from triglycerides for the formation of cancerous cells [6]. Figure 7 shows a summary of potential biomarkers for each group (HC, OS, and M-OS), and Figure 8 summarizes the potential biomarkers of osteosarcoma and metastasis.

In health control (HC, Figure 8a), monoacylglycerols (MAGs), diacylglycerols (DAGs), and triacylglycerols (TAGs) are intermediates and/or biosynthesized in the glycerol–phosphate pathway [70], while in osteosarcoma disease (Figure 8b), a higher overexpression of monoacylglycerol lipases (MAGLs) and diacylglycerol kinases (DGKs) have been reported [28,34]. These enzymes convert MAGs, DAGs, and TAGs to free fatty acids (FFAs) and glycerol during lipolysis in adipocytes [70].

Elevated polyunsaturated fatty acid (PUFA) levels intermediate the four steps of carcinogenesis (initiation, promotion, progression, and metastasis), where the reduced expression of chemokine receptors such as CXCR4 hampers the chemoattraction of metastatic cells (Figure 8c) [71]. While overexpression of phospholipase D (PLD) contributes to higher phosphatidic acids and choline levels [54], elevated cholesterol levels lead to an accumulation of lipids and a protumorigenic state in reprogrammed lipid metabolism [72].

Lipids that were annotated in lipidomic studies via NMR and MS analyses may contribute significantly to clinical studies since they are potential biomarkers. In this sense, biomarkers indicate a patient’s biological state and pathological conditions. Therefore, it is possible to use this information to help develop new diagnosis methods, monitor the cancer stage, understand biochemical processes related to disease, and find new therapies [73,74].

## 4. Conclusions

The results of lipidomics by NMR and MS analyses indicated elevated levels of PUFAs, GPLs, GPCs, and cholesterol in the peripheral blood of patients with osteosarcoma, which followed previous studies reported in the literature. Glycerol levels were decreased, while phosphatidic acid levels were increased in these patients, probably due to the overexpression of DGKs. Moreover, glycerols and choline levels were increased in patients with metastasis at diagnosis, which could be due to phospholipases starting to perform a critical role in the disease development. Future research with different patients and a larger sample size will be necessary to validate these lipids as reliable osteosarcoma biomarkers.

## Figures and Tables

**Table 1:** Assignment of some relevant *m*/*z* features selected by the PLS-DA model (positive ion mode) with increased levels in osteosarcoma (OS) (Figure 6b) and osteosarcoma with metastasis (M-OS) (Figure 6d).

Entry	Experimental *m*/*z* and Class	Theoretical *m*/*z*	Ions	Lipid Assignments	Proposed Formula	Reference/HMDB * ID LipidMaps ID
1	289.2923 (OS and M-OS)	289.2890	[M+H-H_2_O]^+^	14-methylic-en-1- yn-3-ol	C_21_H_38_O	LMFA05000766
2	603.5379 (M-OS)	603.5352	[M+H-H_2_O]^+^ (DAG) or [M-RCOO]^+^ (TAG)	Glycerols	C_39_H_72_O_5_	HMDB0007030, HMDB0007109, HMDB0007137, HMDB0007161, HMDB0007218 [60,61]
3	664.4620 (OS)	664.46	[M+H_2_O+H]^+^	Cer(d18:2/24:1)-Ceramide	C_42_H_79_NO_3_	HMDB0240680 [62]
4	648.4664 (OS and M-OS)	648.4646	[M+H+1]^+^	PA (18:1/14:0)	C_35_H_67_O_8_P	HMDB0114921 LMGP10010882
5	649.4776 (M-OS)	649.4803	[M+H]^+^	PA (16:0/16:0)	C_35_H_69_O_8_P	LMGP10010027
6	758.5730 (OS)	758.5674	[M+H]^+^	PE-NMe (18:1/18:1)	C_42_H_80_NO_8_P	HMDB0010565 LMGP02010338

* HMDB, The Human Metabolome Database.

## Data Availability

The original contributions presented in the study are included in the article/Supplementary Material, further inquiries can be directed to the corresponding author.

## Supplementary Materials

The following supporting information can be downloaded at: https://www.mdpi.com/article/10.3390/metabo14080416/s1, Figure S1: ^1^H-NMR spectra (δ 0.00–8.00) acquired using zg30 pulse sequence; Figure S2: Total Ion Chromatograms (TIC) obtained by ESI(+) LC-MS for lipids; Table S1: Clinical data of patients with bone tumor (osteosarcoma); Table S2: Clinical data of control patients for bone tumor (osteosarcoma); and Table S3: ^1^H-NMR chemical shifts assignments of the metabolites found in lipid extracts of sera from samples used in this lipidomics study.
