# Supplementary material for: Lipidomics by Nuclear Magnetic Resonance Spectroscopy and Liquid Chromatography–High-Resolution Mass Spectrometry in Osteosarcoma: A Pilot Study
Source: Metabolites. 2024 Jul 28;14(8):416. doi: 10.3390/metabo14080416 (PMC11356186; doi:10.3390/metabo14080416)
Supplement: Supplementary file 1 [file metabolites-14-00416-s001.zip › metabolites-3066474-supplementary.pdf]

Supplementary Information

Lipidomics by Nuclear Magnetic Resonance Spectroscopy and Liquid Chromatography-HighResolution Mass Spectrometry in Osteosarcoma: A pilot study

João Guilherme de Moraes Pontes,1 Milka Jadranin,1,2 Márcia Regina Assalin,1,3 Melissa Quintero Escobar,1 Danijela Stanisic,1 Tássia Brena Barroso Carneiro Costa,1 Van Helvoort Lengert,4 Érica Boldrini,5 Sandra Regina Morini da Silva,6 Daniel Onofre Vidal,4 Leticia Huan Bacellar Liu,1 Mariana Maschietto,7,8** Ljubica Tasic1*

[1]Laboratory of Biological Chemistry, Institute of Chemistry, Universidade Estadual de Campinas, Campinas 13083-970, Brazil

[2]University of Belgrade, Institute of Chemistry, Technology and Metallurgy, Department of Chemistry, Njegoševa 12, 11000 Belgrade, Serbia

[3]Embrapa Environment, Jaguariúna, SP, Brazil

[4]Molecular Oncology Research Center (CPOM), Barretos Cancer Hospital, 14784-400, Barretos, Brazil

[5]Barretos Children's Cancer Hospital, 14784-400, Barretos, Brazil

[6]Department of Pathology, Barretos Cancer Hospital, 14784-400, Barretos, Brazil

[7]Brazilian Biosciences National Laboratory (LNBio), Brazilian Center for Research in Energy and Materials (CNPEM), 13083-100, Campinas, Brazil

[8]Current address: Research Center, Boldrini Children's Hospital, 13083-884, Campinas, SP, Brazil

*Correspondence: ljubica@unicamp.br

Table S1. Clinical data of patients with bone tumor (osteosarcoma)

Nº	Age (years)	Gender	Histologic type	Stage	Metastasis (at diagnosis)	Status
1	17	M	Telangiectatic	NR	NR	Alive without disease
2	18	F	Osteoblastic	II	Absent	Death due to another disease
3	35	M	Osteoblastic	I	Present	NR
4	20	M	Osteoblastic	I	Absent	Death due to cancer
5	14	M	Chondroblastic	I	Absent	Death due to another disease
6	15	F	Osteoblastic	I	Present	Death due to another disease
7	17	M	Osteoblastic	II	Absent	Death due to cancer
8	43	M	Osteoblastic	II	Absent	NR
9	17	M	Osteoblastic	I	Present	Death due to cancer
10	17	F	Fibroblastic	I	Absent	Alive without disease
11	18	F	Osteoblastic	II	Present	Death due to cancer
12	12	F	Osteoblastic	II	Absent	Death due to cancer
13	13	M	NR	NR	Present	Alive without disease
14	19	F	Osteoblastic	II	Present	Death due to cancer
15	16	F	Telangiectatic	I	Absent	Death due to another disease
16	29	F	Osteoblastic	I	Absent	NR
17	19	F	Periosteal	NR	Absent	NR

18	12	M	Osteoblastic	I	Absent	Alive without disease
19	NR	NR	NR	NR	NR	NR
20	NR	NR	NR	NR	NR	NR
21	NR	NR	NR	NR	NR	NR

*All samples were duplicated and treated like different samples to improve statistical analysis except for samples marked as NR; **NR, non-reported data - NR samples were classified as belonging to the OS group. ***Stage cancer is classified following osteosarcoma diagnosis before metastasis. M-OS samples were collected later.

Table S2. Clinical data of control patients for bone tumor (osteosarcoma)

No.	Age (years)	Gender	Status	No.	Age (years)	Gender	Status
22	4	M	Cancer free control	26	6	F	Cancer free control
23	9	M	Cancer free control	27	32	F	Cancer free control
24	4	F	Cancer free control	28	8	F	Cancer free control
25	36	F	Cancer free control	29	5	M	Cancer free control

*All samples were duplicated and treated like different samples to improve statistical analysis.

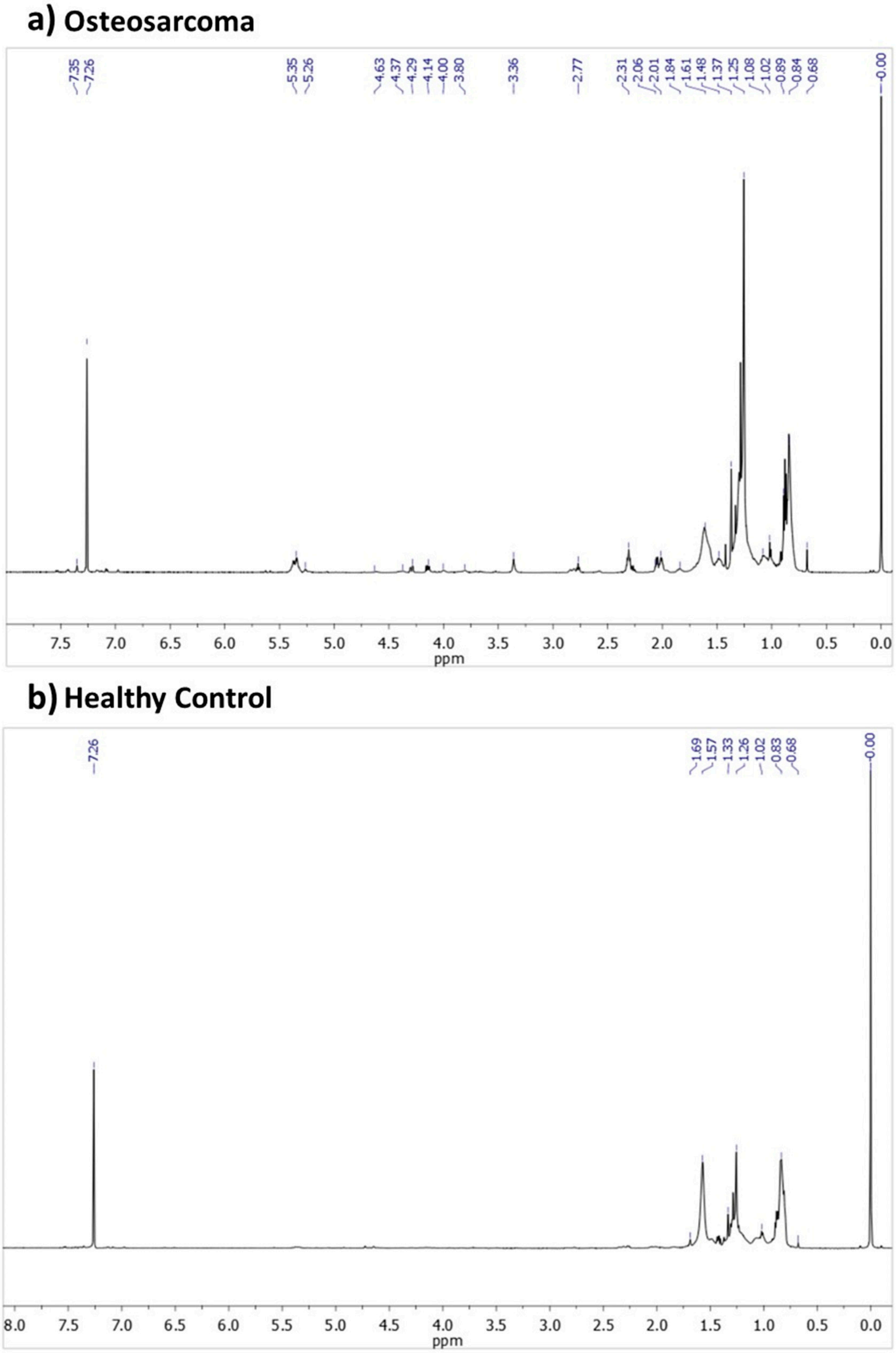

Figure S1. ^{1}H-NMR spectra (**δ** 0.00–8.00) acquired using *zg30* pulse sequence (a) representative serum lipid extracts of osteosarcoma patients and (b) healthy control.

Table S3. ^{1}H-NMR chemical shifts assignments of the metabolites found in lipid extracts of sera from samples used in this lipidomics study

Entry	Metabolite	δ ^{1}H and multiplicity* (Databases)	δ ^{1}H (Experimental)	References HMDB BMRB
1	Cholesterol	0.64 (s), 0.79 (d), 0.91–0.93 (m), 0.99 (s), 1.11–1.16 (m), 1.49–1.52 (m), 2.44 (dd), 3.67–3.70 (m), 5.31 (dd)	0.68, 0.91, 1.02, 3.77–3.83 5.35	HMDB0000067 bmse000961
2	Fatty acyl chains (FA)/ polyunsaturated fatty acids (PUFAs)	0.90 (t), 1.28–1.31 (m), 1.37 (m), 1.58 (m), 2.04 (m), 2.24 (m), 2.81 (m), 5.31 (m), 5.60 (m)	0.87, 1.25–1.38, 1.62, 2.02, 2.30, 2.79, 4.60, 5.37, 5.61	Reference 30
3	Choline (Cho)	3.19 (s), 3.51 (dt), 4.05 (t)	3.20, 3.52, 4.04	HMDB0000097 bmse000953
4	Glycerophosphocholines (GPC)	3.20 (s), 3.59–3.68 (m), 3.84–3.95 (m), 4.29–4.32 (m)	3.20, 3.61–3.70, 3.80–3.84, 4.32–4.37	HMDB0000086
5	Phosphoethanolamines (PE)	3.26 (m), 3.92 (m)	3.93	Reference 22
6	Monoacylglycerols (MAG)	2.04 (s), 2.32 (m), 2.80 (t), 3.72 (m), 4.14 (m), 5.35 (m)	2.31, 2.77, 3.71, 4.19, 5.34	Reference 17
7	Diacylglycerols (DAG)	3.72 (brs), 4.13–4.18/4.29–4.31 (dd), 5.08(s), 5.26 (m), 5.28–5.40 (m)	3.72, 4.16, 5.07	Reference 23
8	Glycerophospholipids (GPL)	1.23–1.66 (m), 2.55 (m), 5.19 (brs), 5.32 (brs)	2.55–2.60, 2.80, 5.21, 5.34	Reference 31
9	Triacylglycerol (TAG)	4.33 (dd), 5.28 (m)	4.16, 5.26	Reference 22

*s, singlet; brs, broad singlet; d, doublet; t, triplet; q, quartet; m, multiplet; dd, doublet of doublets; dt, doublet of triplets.

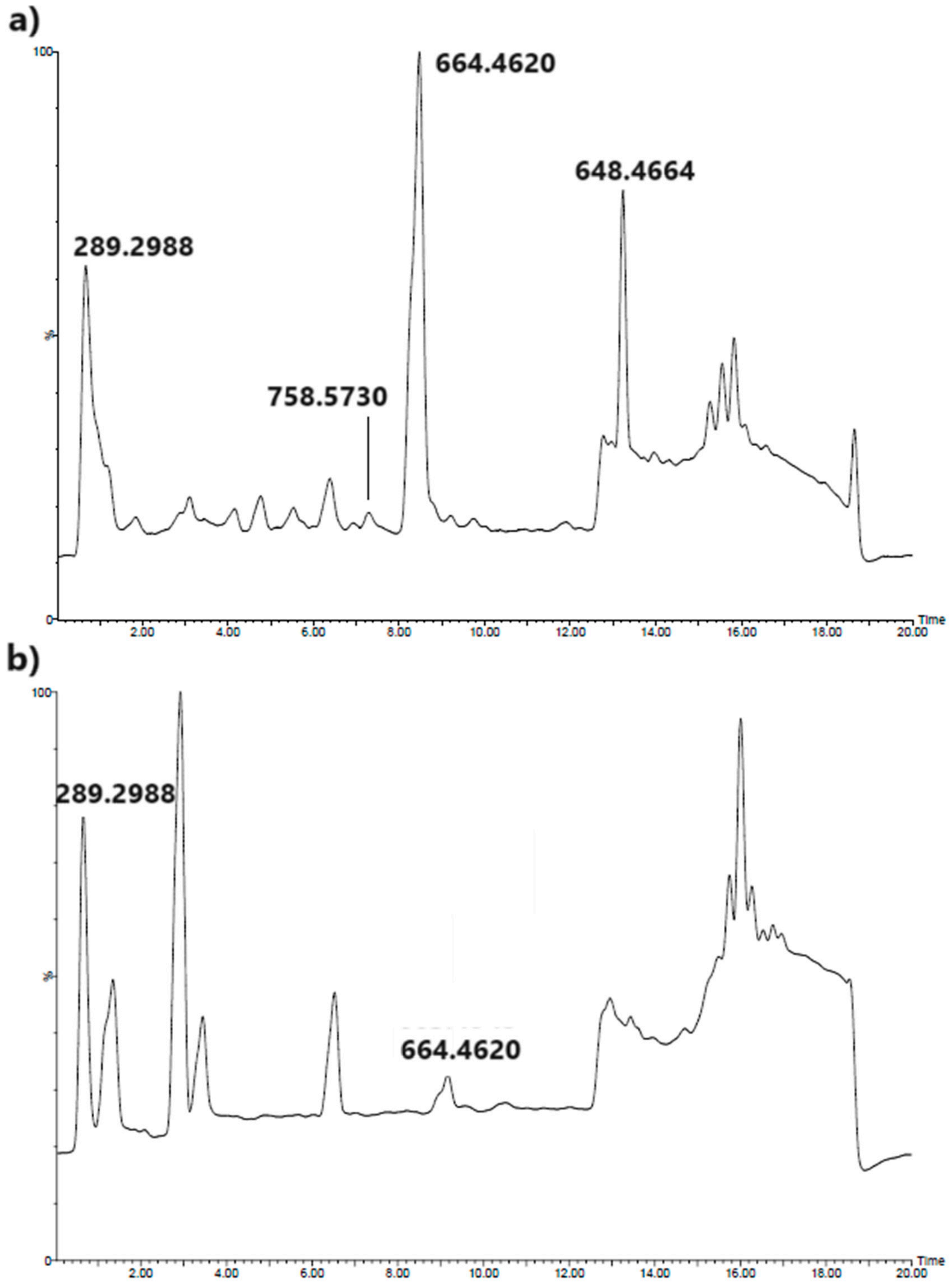

Figure S2. Total Ion Chromatograms (TIC) obtained by ESI(+) LC-MS for lipids of (a) osteosarcoma patient and (b) healthy control.
